# What are the sympatric mechanisms for three species of terrestrial hermit crab (*Coenobita rugosus*, *C*. *brevimanus*, and *C*. *cavipes*) in coastal forests?

**DOI:** 10.1371/journal.pone.0207640

**Published:** 2018-12-12

**Authors:** Chia-Hsuan Hsu, Marinus L. Otte, Chi-Chang Liu, Jui-Yu Chou, Wei-Ta Fang

**Affiliations:** 1 School of Forestry and Resource Conservation, National Taiwan University, Taipei City, Taiwan, ROC; 2 Wet Ecosystem Research Group, Department of Biological Sciences, NDSU Dept. 2715, North Dakota State University, Fargo, North Dakota, United States of America; 3 Department of Biology, National Changhua University of Education, Changhua, Taiwan, ROC; 4 Graduate Institute of Environmental Education, National Taiwan Normal University, Taipei City, Taiwan, ROC; University of California, UNITED STATES

## Abstract

Terrestrial hermit crabs play a significant role in coastal ecology. For example, as seed dispersers and debris scavengers in coastal forests, they accelerate the decomposition of organic substances. In the Indo-Pacific Ocean, *Coenobita rugosus*, *C*. *brevimanus*, and *C*. *cavipes* are the three most common species of terrestrial hermit crab. Because the mechanisms that contribute to the sympatry of these three species of crab have not been identified, this study investigated the three most likely explanations: niche differences, competition, and predation. The results showed that the three species displayed niche differences in terms of seasonal activity, habitat, utilization of shells, and food preference, suggesting that competition for resources is avoided. The habitat of terrestrial hermit crabs in Taiwan is closely associated with that of humans. Our study helps improve our understanding of the ecology of terrestrial hermit crabs and their conservation.

## 1. Introduction

Terrestrial hermit crabs are widely distributed in tropical and subtropical regions. They play major roles in ecosystems and substantially contribute to nutrient cycling and seed dispersal in coastal forests [1, 2]. Terrestrial hermit crabs are scavengers of terrestrial debris and several species may occur sympatrically in coastal forests [3–5]. However, knowledge about sympatry of terrestrial hermit crabs, and information to underpin their conservation and management is lacking. Sympatry means two species or population exist in the same geographic area and usually meet one another. When two similar species live in the same region, they may face competition for resources. To avoid this, species may exhibit niche differences, which reduces habitat overlap and decreases intensity of competition [6]. Therefore, we investigated the importance of different mechanisms that may explain the sympatry of the terrestrial hermit crabs, *Coenobita rugosus*, *C*. *brevimanus*, and *C*. *cavipes*, regarding (1) ecological niche differences (shells, food, seasonal activity), (2) ecological niche overlap and competition for resources, and (3) ecological niche overlap and predation.

In Taiwan, five species of terrestrial hermit crab have been recorded: *C*. *rugosus*, *C*. *brevimanus*, *C*. *cavipes*, *C*. *violascens*, and *C*. *purpureus* [5, 7–9], but the latter two species are not abundant enough for inclusion in our study. *C*. *rugosus*, *C*. *brevimanus*, and *C*. *cavipes* are abundant in Taiwan and distributed throughout the Indo-Pacific Ocean.

For hermit crabs, niche differences may relate to habitat, shell type, diet, or seasonal activity. If no niche differences occur, the crabs must compete for resources. Although distance from shore and elevation may be independent, because further distance from the sea does not necessarily mean higher elevation [10], our study did not have enough data points to test both distance from shore and elevation separately. In our study, distance from shore in most cases also meant higher elevation. We therefore only tested distance from shore as a measure of ‘habitat’. Food is a limited resource, and organisms therefore frequently compete for it. Terrestrial hermit crabs use their sense of smell to search for food. In the same habitat, different foraging behaviors or olfactory abilities are sufficient to cause ecological niche differences and ensure the joint survival of multiple species [11]. Although they are scavengers and have seemingly unselective food choices, they exhibit clear preferences [3, 10, 12]. They ingest fruit, fallen leaves, seeds, animal excretions, and animal carcasses [3, 13, 14]. Tran (2013) found that the olfactory capacities of the marine hermit crab species *Clibanarius digueti* and *Paguristes perrieri* differ, resulting in foraging niche differences and sympatric survival in the same habitat [15]. Morrison (2002) found that the terrestrial hermit crab *Coenobita clypeatus* has a competitive relationship with ants regarding food, but because of temporal variation in foraging activity and complementary foraging strategies, competition was limited [16].

Shells are in limited supply but are indispensable to the basic survival of hermit crabs. During the process of growth, hermit crabs continue to change shells to suit their body size. The main functions of hermit crab shells are 1) to prevent loss of water; 2) to protect against predatory attacks; and 3) to provide enough room to hold the eggs of female crabs [17–19]. Placing 12,000 empty shells in an intertidal zone of rocks significantly increased the density of the hermit crab *Pagurus hirsutiusculus* in comparison with areas where empty shells were not placed [20]. This indicates that shells are an important limiting factor in the wild. Given the scarcity of shells, fighting for shells is a common occurrence [21, 22]. Several studies have shown that different species of hermit crabs will exhibit different preferences for shells as well [20, 23–25], which reduces competition for this resource. Terrestrial hermit crabs often locate shells by smell [26].

The aim of our study therefore was to investigate the sympatric mechanisms for three species of terrestrial hermit crab (*C*. *rugosus*, *C*. *brevimanus*, and *C*. *cavipes*). We expected that differences between the species regarding seasonal activity, habitat preference, availability and type of shells, and food preferences would all to some extent contribute to the sympatry of these species.

## 2. Materials and methods

About this field research, we got the research permission and granted permission from Kenting National Park Headquarter and issued by the Ministry of Science and Technology MOST (106-2119-M-003-003-). The authority, Kenting National Park Headquarter, responsible for protected area of land or sea, as well as concerned with protection of wildlife. We confirmed that the field studies did not involve endangered or protected species.

### 2–1 Field surveys

**Fig 1:**
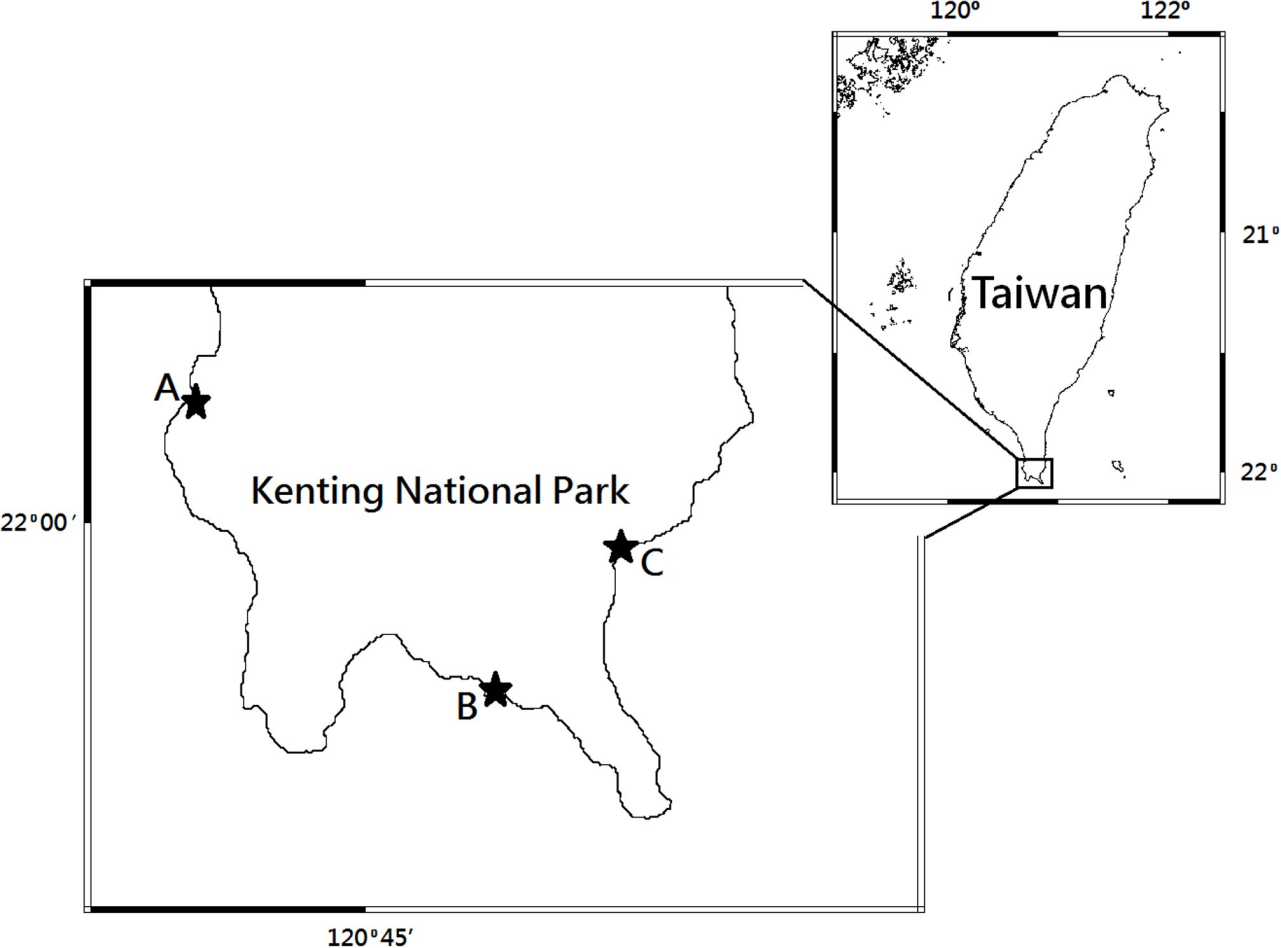
Sampling sites in this study. (A) Houwan; (B) Qingwashi; (C) Gangkou.

The research location was at Kenting National Park in southern Taiwan (21°56’53.6"N, 120°46’49.6"E). It has a tropical climate and is rich in uplifted coral reef, tropical rainforest, coastal forest, and sand beaches, and is habitat for *C*. *rugosus*, *C*. *brevimanus*, *C*. *cavipes*, *C*. *violascens* and *Birgus latro*. The survey methods used for the preliminary investigation were sampling and measurement of crabs that had been caught and collected. The survey locations were: (A) Houwan, (B) Qingwashi, and (C) Gangkou, located in the east, west, and south, respectively (Fig 1).

**Fig 2:**
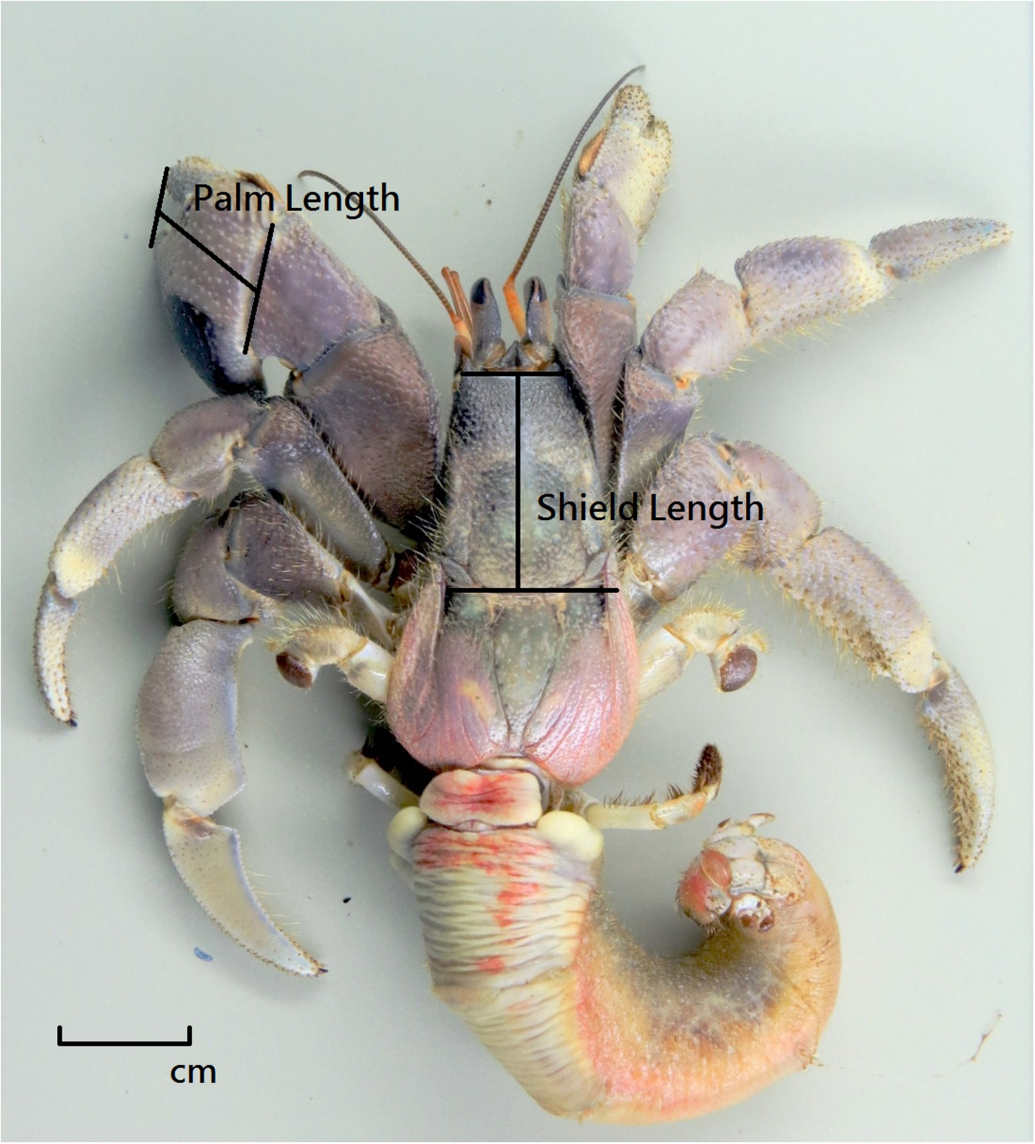
Shield length and palm length of a hermit crab (*C*. *rugosus*).

Rice bran was used to lure and catch the crabs. In preparation for this, raw rice bran was fired at a low heat for about five minutes until it became fragrant [10]. Before the sun set, possible terrestrial hermit crab habitats were investigated (such as sandy beaches, coastal forests, or reef seams), and the rice bran placed (about 100 ml) one hour before sunset (about 17:30 in October and January; 18:00 April and July). GPS (Google Earth) was used to record latitude and longitude. After sunset, the hermit crabs were collected in a bucket. The species were recorded, and a ruler (minimum unit: mm) was used to measure the shield length, the palm length, the species of shell, and the shell width. No animals were sacrificed during any part of this study. In these species, the left chelae are larger than their right chelae and serve as lids for blocking their shells [27]. We used shield length as the indicator for hermit crab body size (Fig 2), and this was measured when possible. A few hermit crabs did not come out of their shells, which meant the shield length could not be measured. In these cases, we estimated shield length by regression analysis, based on palm length. The conversion equations were [5]:

C.rugosus:Shieldlength(SL,cm)=Palmlength(PL,cm)*0.88+0.02

C.cavipes:Shieldlength(SL,cm)=Palmlength(PL,cm)*0.7+0.048

C.brevimanus:Shieldlength(SL,cm)=Palmlength(PL,cm)*0.5+0.24

### 2–2 Seasonal activity

Species investigations were conducted at the three locations once during 1st-3rd of April, 8th-10th of July, 6th-9th of October in 2017, and 29th-31st of January in 2018. Percentages by season and species were then used to explore whether temporal niche differences was observed, using the following equation for each species: The quantity of catch for the season/the total quantity of catch throughout the year x 100%. The Chi-squared test was used to ascertain whether different species showed differences in seasonal activity.

### 2–3 Habitat

After measuring the latitude and longitude of each sample site, the data were imported into Google Maps to calculate the distance between traps. The Chi-squared test was then used to ascertain whether different species showed niche differences in terms of their habitat (distance from the sea shore and elevation).

### 2–4 Shell preference

The mature animals differ in body size between species, which may be one reason to select different species of shells. Such differences may be displayed earlier in development and we therefore, for this comparison, selected crabs in sizes in the range of overlap between the three species of 0.7–1.3cm. Only individual hermit crabs whose shells fitted their entire body well, without any soft tissues exposed and able to close the opening with their chelae, were used for this analysis. The species of shell used by the crabs were determined. The Kruskal-Wallis was then used to assess differences in preferences between the three crab species of similar body size for shells of the same Family. Additionally, shell width as a measure of shell size was included in Kruskal-Wallis and Mann-Whitney tests to determine whether the three species displayed significant differences regarding their preferences for shell size in the same body size of three species [28].

### 2–5 Food preference

We used a field experiment to test preference for food. The experimental steps were as follows:

1. Fish (Pacific saury, *Cololabis saira*) and banana were used as the experimental treatment following Burggren and McMahon (1988) [1]
2. The foods were placed into separate 30 × 30cm laundry bags.
3. The laundry bags were placed at the same sampling sites in the thee sampling locations and the distance between each of the two laundry bags was 5 meters.
4. After 1 hour, the terrestrial hermit crab species foraging on the laundry bags were collected and the quantity of each species recorded.
5. Fisher’s exact test was used to determine whether significant differences existed between the terrestrial hermit crab species in terms of their food preferences.

## 3. Results and discussion

### 3–1 Seasonal activity

**Fig 3:**
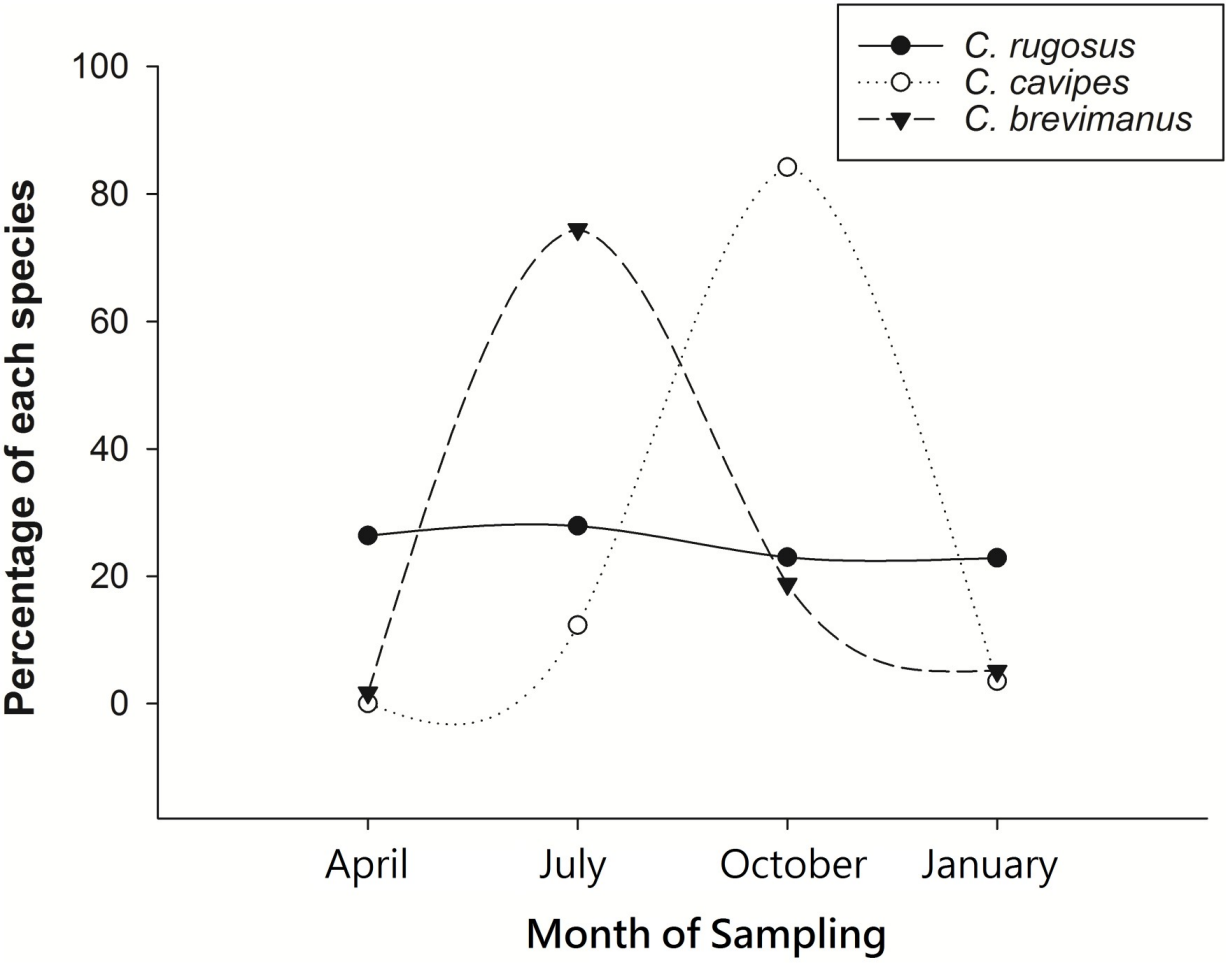
Relative numbers (% of total numbers captured for this study) of species of terrestrial hermit crab during April, July, October, 2017, and January, 2018. Significance of differences as tested by χ^2^-Test are given in Table 1.

The total number of *C*. *rugosus* collected was 1690, *C*. *brevimanus* 176, and *C*. *cavipes* 57. As a measure of seasonal activity, the number of hermit crabs of each species collected each season were divided by the total number of hermit crabs and expressed as a percentage (Fig 3). *C*. *rugosus* showed the least variation, representing 34% in April, 36% in July, 30% in October, and 23% in January. The numbers of *C*. *brevimanus* were 1.8% in April, 78% in July, 20% in October, and 5% in January. For *C*. *cavipes* the percentages were 0% for April, 13% for July, 97% for October, and 4% for January.

Statistical analysis using the χ^2^-Test (Table 1) indicated that the months of highest activity showed significant differences, with *C*. *brevimanus* having peak activity in July, and *C*. *cavipes* in October. Those peak activities were much higher than the activity of *C*. *rugosus*, which was more-or-less constant throughout the year.

**Table 1:** Differences between species in the seasonal activity (April, July, October, 2017, and January 2018). *P* = probability (χ^2^-test).

**Species/Month**	**April**	**July**	***P***
	*C*. *cavipes* and *C*. *brevimanus* v.s *C*. *rugosus*		
*C*. *rugosus*	445	471	
*C*. *cavipes*	0	7	<0.05
*C*. *brevimanus*	3	131	<0.001
	*C*. *cavipes* v.s *C*. *brevimanus*		
*C*. *cavipes*	0	7	
*C*. *brevimanus*	3	131	0.346
**Species/Month**	**July**	**October**	***P***
	*C*. *cavipes* and *C*. *brevimanus* v.s *C*. *rugosus*		
*C*. *rugosus*	471	388	
*C*. *cavipes*	7	48	<0.001
*C*. *brevimanus*	131	33	<0.001
	*C*. *cavipes* v.s *C*. *brevimanus*		
*C*. *cavipes*	7	48	
*C*. *brevimanus*	131	33	<0.001
**Species/Month**	**October**	**January**	***P***
	*C*. *cavipes* and *C*. *brevimanus* v.s *C*. *rugosus*		
*C*. *rugosus*	388	386	
*C*. *cavipes*	48	2	<0.001
*C*. *brevimanus*	33	9	<0.001
	*C*. *cavipes* v.s *C*. *brevimanus*		
*C*. *cavipes*	48	2	
*C*. *brevimanus*	33	9	<0.05

*C*. *rugosus* also showed stable numbers throughout the year, and the total number far exceeded those of the other two species, either because of their specific reproductive strategy or the fact that the other two species are vulnerable to capture by natural enemies including humans for use as fishing bait, and for the commercial pet market in Taiwan [29], and around the world.

### 3–2 Niche differences for habitat

In terms of habitat niche differences, distance from the sea shore was assessed. According to Burggren and McMahon (1988), *C*. *rugosus* is typically found near the shore but inland from the beach, though never more than about 100 m from high tide [1]. Therefore, we used the 50 and 100m as the cut-off point. We used the chi-squared test to analyze whether distance from the sea shore was different between the species of crabs. The results showed that at a distance of 50 m from the shore, the numbers of *C*. *rugosus* and *C*. *cavipes* were significantly different. *C*. *rugosus* was closer to the shore (χ^2^ = 42.17, p < 0.001). The numbers of *C*. *rugosus* and *C*. *brevimanus* also differed significantly, and *C*. *rugosus* was closer to the shore (χ^2^ = 149.43, p < 0.001). The numbers for *C*. *cavipes* and *C*. *brevimanus* did not differ significantly (χ^2^ = 3.055, p = 0.08) (Table 2).

**Table 2:** Differences in horizontal distance from the shore for three terrestrial hermit crab species (basis: 50 and 100 meters). *P* = probability (χ^2^-test).

	**Horizontal distance from the shore**		
**Species**	**< 50m**	**>50m**	***P***
	*C*. *cavipes* and *C*. *brevimanus* vs *C*. *rugosus*		
*C*. *rugosus*	748	734	
*C*. *cavipes*	3	53	< 0.001
*C*. *brevimanus*	1	167	< 0.001
	*C*. *brevimanus* vs *C*. *cavipes*		
*C*. *cavipes*	3	53	
*C*. *brevimanus*	1	167	0.08
	**Horizontal distance from the shore**		
**Species**	**< 100m**	**>100m**	***P***
	*C*. *cavipes* and *C*. *brevimanus* vs *C*. *rugosus*		
*C*. *rugosus*	1447	35	
*C*. *cavipes*	17	39	< 0.001
*C*. *brevimanus*	5	163	< 0.001
	*C*. *brevimanus* vs *C*. *cavipes*		
*C*. *cavipes*	17	39	
*C*. *brevimanus*	5	163	< 0.001

At 100 m from the sea shore the numbers of *C*. *rugosus* and *C*. *cavipes* were significantly different as well (χ^2^ = 518.75, p < 0.001) and *C*. *rugosus* was closer to the sea shore. The same applied to *C*. *rugosus* and *C*. *brevimanus*, and again *C*. *rugosus* was significantly closer to the sea shore (χ^2^ = 1271.5, p < 0.001). At this distance, the numbers of *C*. *cavipes* and *C*. *brevimanus* were significantly different as well (χ^2^ = 32.53, p < 0.001) (Table 2). Therefore, C. *rugosus* was closest to the shore, followed by *C*. *cavipes*, then *C*. *brevimanus*, which was farthest from the shore.

**Fig 4:**
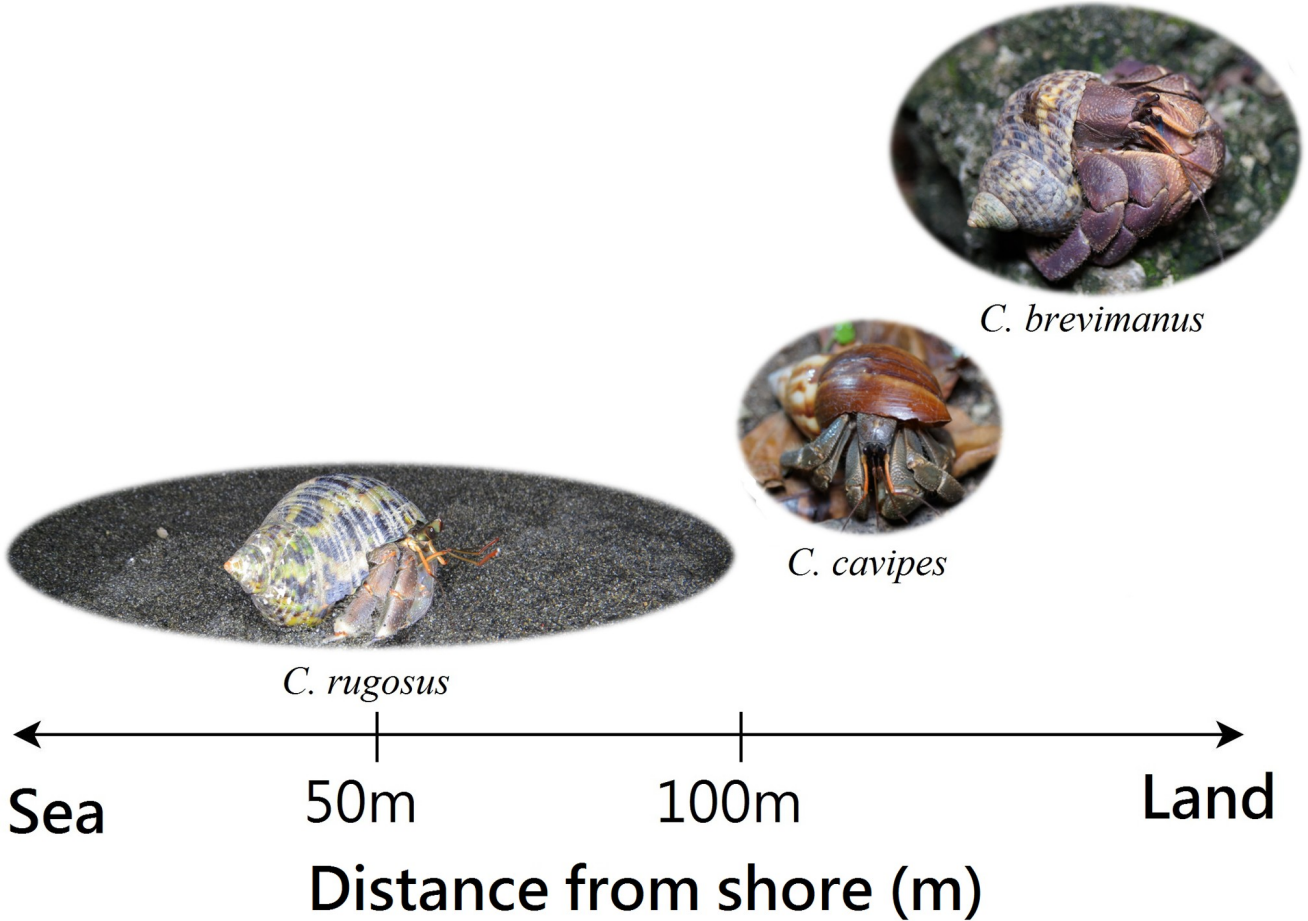
Distribution of three terrestrial hermit crab species in terms of distance from the shore. Widths of ovals indicated approximate ranges.

We can see the distribution of three terrestrial hermit crab species in terms of distance from the shore in Fig 4. Additionally, the reason why all three species of terrestrial hermit crabs are sometimes seen on sandy beaches is that there may be an overlapping critical region, or it may be that they have released their larvae by the shore during the breeding season and have yet to return to their original habitat. Nevertheless, this study found that these species all differ in terms of their preferred habitat.

### 3–3 Niche differences for shell resource

Shells are vital for terrestrial hermit crabs, because they protect the soft tissues of the animal. This study also explored if different terrestrial hermit crab species with bodies of the same size range showed niche differences in terms of shells and suitable opening sizes. The shells carried by individual hermit crabs as well as the relationship between body size and shell were analyzed to determine each species’ preferred family of shells.

Regarding shell selection, the mollusk shell preferences of the three terrestrial hermit crabs differed as follows: *C*. *rugosus* preferred the shells of Family Muricidae and Neritidae; *C*. *cavipes* preferred the shells of Family Achatinidae and Ampullariidae; and *C*. *brevimanus* preferred the shells of Family Babyloniidae and Turbinidae (Table 3). The results showed that *C*. *cavipes* had a notable preference for relatively large shells and preferred terrestrial mollusk species such as the African Snail (*Achatina fulica*) and the freshwater mollusk like Apple Snail (*Pomacea canaliculata*). Possible reasons for this are that large shell widths in marine snail species are uncommon, or that *C*. *cavipe*s dwells predominantly on land and can only obtain terrestrial mollusk shells.

**Table 3:** Differences in families of shells preferred by the three species in the overlapping body size range of 0.7–1.3 cm shield length. Entries are ordered according to highest percentage for each species. The same color represents the same family of shells.

Species	*C*. *rugosus* (n = 97)	*C*. *brevimanus* (n = 45)	*C*. *cavipes* (n = 21)
**Sequence of shell preference**	Muricidae (38.1%)	Babyloniidae (55.7%)	Achatinidae (64.7%)
	Neritidae (23.1%)	Turbinidae (18%)	Ampullariidae (17.6%)
	Others (14.4%)	Others (11.5%)	Babyloniidae (8.8%)
	Babyloniidae (10%)	Muricidae (4.9%)	Others (5.8%)
	Turbinidae (8.1%)	Melongenidae (4.9%)	Muricidae (2.9%)
	Achatinidae (3.1%)	Ampullariidae (1.6%)	
	Conidae (1.9%)	Achatinidae (1.6%)	
	Ampullariidae (1.3%)	Neritidae (1.6%)	

Significant differences existed between crab species and preference for shell species (p < 0.01, Kruskal-Wallis). The three species of crabs also preferred significantly different shell width (Table 4, Mann-Whitney test). The species preferring the largest shell widths were *C*. *cavipes* and *C*. *rugosu*, followed by *C*. *brevimanus*.

**Table 4:** Differences in shell preference of three species of terrestrial hermit crabs with overlapping body size (0.7–1.3 cm, shield length) and differences in shell size *P* = probability (Mann-Whitney test).

Species	Count	Mean rank.	*P*
*C*. *rugosus*	97	55.4	
*C*. *cavipes*,	21	69.2	0.11
*C*. *rugosus*	97	55.0	
*C*. *brevimanus*	45	79.1	0.001
*C*. *cavipes*,	21	43.0	
*C*. *brevimanus*	45	27.1	0.002

This observation makes sense, because the left palms (chela) of the three species of crab differ in size when comparing crabs with the same overall body size [5]. The chela is the lid used to block the shell and protect the body once a terrestrial hermit crab retracts into its shell. Therefore, different species have different preferences for shells. In terms of shells, such preferences are a way to prevent and limit competition for resources [17, 18, 22, 30]. Crabs carrying relatively small shells may be limited in growth and may be at a higher risk of death due to dehydration or predation [17–19, 30].

## 4. Niche differences for food preference

To explore food preference niche differences among the three species of crabs, Pacific saury was used to represent fish, whereas banana was the representative fruit. Fisher’s exact test showed that *C*. *rugosus* and *C*. *cavipes* exhibited no difference in food preference (p = 0.057, Fisher’s exact test); nor was there any difference between C. *rugosus* and *C*. *brevimanus* (p = 0.271, Fisher’s exact test). However, differences existed between *C*. *brevimanus* and *C*. *cavipes* in that C. *brevimanus* preferred fish while *C*. *cavipes* preferred fruit (p < 0.01, Fisher’s exact test) (Table 5). We can speculate that because *C*. *rugosus* dwells in areas nearer the sea, it has access to a great diversity in food sources, ranging from seaweed to the bodies of dead fish; *C*. *cavipes* dwells in inland coastal forest areas, with probably better access to plants, fruit, or seeds. However, this logic does not explain why *C*. *brevimanus* prefers fish. One possible reason may be that various types of terrestrial animal carrion are far from the sea shore.

**Table 5:** Differences in dietary preferences of three terrestrial hermit crab species *P* = probability (Fisher’s exact test).

Species /Food treatment	Banana	Saury	*P*
	*C*. *cavipes* and *C*. *brevimanus* vs *C*. *rugosus*		
*C*. *rugosus*	9	14	
*C*. *cavipes*	8	2	0.057
*C*. *brevimanus*	2	9	0.271
	*C*. *brevimanus* vs *C*. *cavipes*		
*C*. *cavipes*	8	2	
*C*. *brevimanus*	2	9	< 0.01

**Fig 5:**
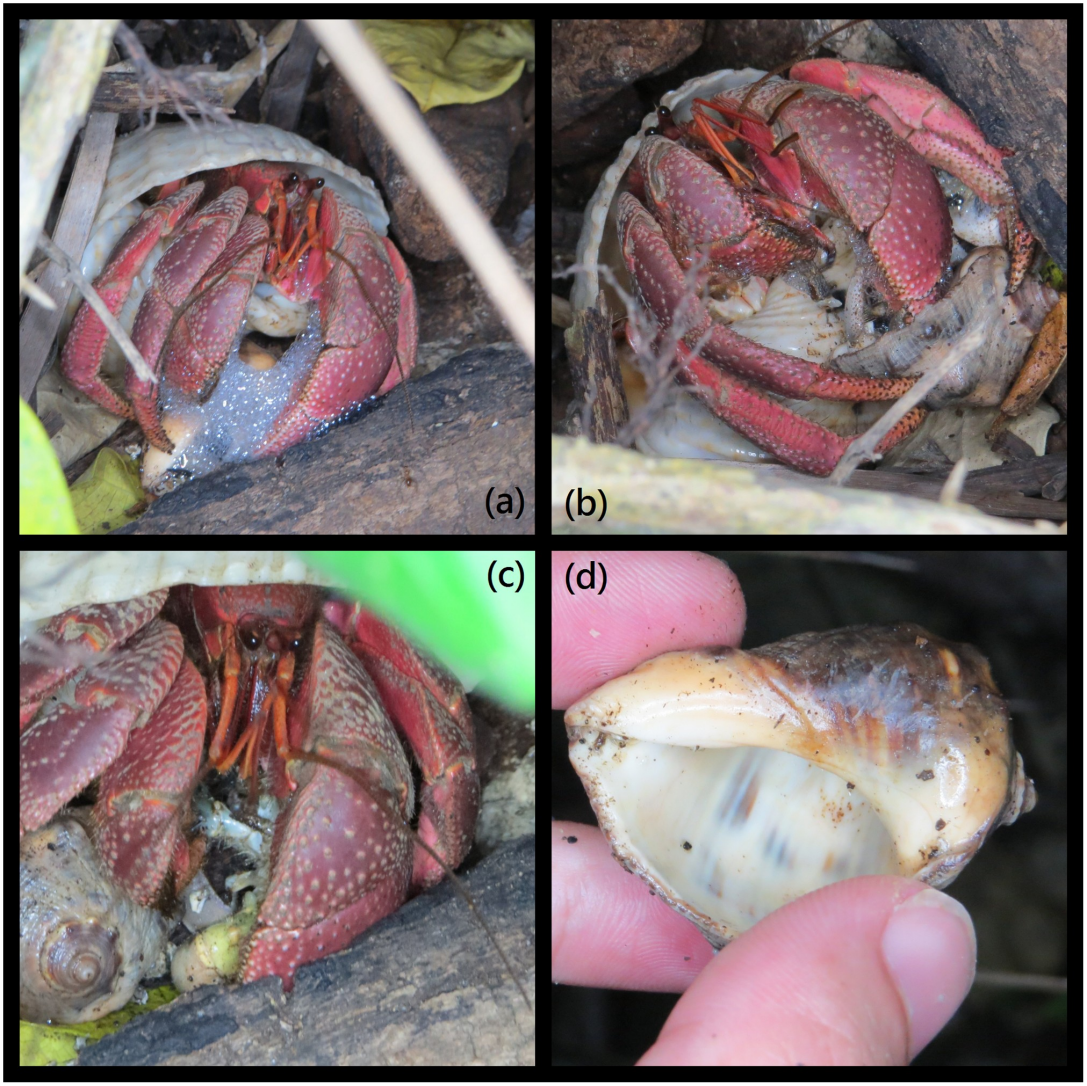
*C*. *brevimanus* preying on *C*. *rugosus* (in situ photograph). (a): *C*. *brevimanus* showing predatory and bubble blowing behavior; (b): Forcing *C*. *rugosus* out using the right chela; (c): *C*. *rugosus* being eaten (the abdomen is displayed); (d): Only the shell remains after one hour.

In addition, we observed *C*. *brevimanus* preying on *C*. *rugosus* (Fig 5), suggesting that *C*. *brevimanus* is not only a scavenger but also a predator. Moreover, through observation, it was noted that *C*. *brevimanus* displayed bubble blowing when preying and feeding on *C*. *rugosus*. Whether this behavior is special or occurs occasionally during preying and feeding requires more in-depth research. Other studies have also shown that *Birgus latro*, the largest species in the Family Coenobitidae, is not only a scavenger but also a predator. It hunts other terrestrial crabs, birds and rats [31–33]. Ascertaining whether the predatory stress of *C*. *brevimanus* causes habitat niche differences among the other two species of terrestrial hermit crabs will require further research. One avenue to explore would be a gut content analysis study to determine exactly what the crabs were eating.

This study explored the sympatric relationship between three common species of terrestrial hermit crab in the Kenting area in Taiwan. Previously, we believed that the species, found in coastal forests, sandy beaches, and inland areas, dwelled in the same area, competing for the same resources. However, this study found that seasonal activity, preferences for habitat, shell species, and shell size, and dietary habits showed considerable differences between the species.

## 5. Conclusion

This study explored the sympatric relationship between three common species of terrestrial hermit crab in the Kenting area in Taiwan. Previously, we believed that the species, found in coastal forests, sandy beaches, and inland areas, dwelled in the same area, competing for the same resources. However, this study found that seasonal activity, preferences for habitat, shell species, and shell size, and dietary habits showed considerable differences between the species.

The results of this study are valuable, not only for our understanding of the ecology and behavior of terrestrial hermit crabs, but also to conservation-related action, policy, and education, as well as to raise environmental literacy. Terrestrial hermit crabs are widely sold in pet markets, and poaching is a big problem. In addition, as the distribution of these hermit crabs in Taiwan is close to urban areas, habitat destruction is an issue of concern. Now that we better understand the habitat preferences for these three species of hermit crabs we can enhance the policies and management in Kenting National Park, Taiwan, and across their entire natural range.

## Supporting information

S1 File Relative numbers (% of total numbers captured for this study) of species of terrestrial hermit crab during April, July, October, 2017, and January, 2018. (PDF)

S2 File Differences in horizontal distance from the shore for three terrestrial hermit crab species (basis: 50 and 100 meters). (PDF)

S3 File Differences in vertical distribution of three terrestrial hermit crab species. (PDF)

S4 File Differences in shell preference of three species of terrestrial hermit crabs with overlapping body size (0.7–1.3 cm, shield length) and differences in shell size. (PDF)

S5 File Differences in dietary preferences of three terrestrial hermit crab species. (PDF)
