# Supplementary material for: What are the sympatric mechanisms for three species of terrestrial hermit crab (Coenobita rugosus, C. brevimanus, and C. cavipes) in coastal forests?
Source: PLoS One. 2018 Dec 12;13(12):e0207640. doi: 10.1371/journal.pone.0207640 (PMC6291072; doi:10.1371/journal.pone.0207640)
Supplement: S1 File — (PDF) [file pone.0207640.s001.pdf]

S1. Relative numbers (% of total numbers captured for this study) of species of terrestrial hermit crab during April, July, October, 2017, and January, 2018.

**Species**	**April, 2017**	**July, 2017**	**October, 2017**	**January, 2018**	**Total**
C. rugosus	445	471	388	386	1690
C. cavipes	0	7	48	2	57
C. brevimanus	3	131	33	9	176
