# Supplementary material for: What are the sympatric mechanisms for three species of terrestrial hermit crab (Coenobita rugosus, C. brevimanus, and C. cavipes) in coastal forests?
Source: PLoS One. 2018 Dec 12;13(12):e0207640. doi: 10.1371/journal.pone.0207640 (PMC6291072; doi:10.1371/journal.pone.0207640)
Supplement: S2 File — (PDF) [file pone.0207640.s002.pdf]

S2. Differences in horizontal distance from the shore for three terrestrial hermit crab species (basis: 50 and 100 meters).

Horizontal distance(m)	*C. rugosus*(count)	*C. cavipes*	*C. brevimanus*
52.4	9		
64.74	20		
24.35	6		
21.22	2		
31.82	90		
52.4	2		
64.74	20	4	
24.35	63		
21.22	131		
31.82	71		1
52.4	30	8	2
64.74	8	1	1
24.35	84	2	
21.22	108		
31.82	93	1	
282		31	2
17.7	17		
100			
169.4			
211.2			
265.8			
176.9			3
17.7	21		
100			8
169.4			27
211.2			10
265.8			24
176.9			60
17.7	62		
100			
169.4			9
211.2		1	5
265.8			5
176.9			9

445.01			
392			
53	78		
79.2	72		
82	151		
445.01	5	1	1
392	27	2	
53	97		
79.2			
82	34		
445.01	3	4	
392			
53	125		
79.2			
82	53	1	1
