# Supplementary material for: What are the sympatric mechanisms for three species of terrestrial hermit crab (Coenobita rugosus, C. brevimanus, and C. cavipes) in coastal forests?
Source: PLoS One. 2018 Dec 12;13(12):e0207640. doi: 10.1371/journal.pone.0207640 (PMC6291072; doi:10.1371/journal.pone.0207640)
Supplement: S3 File — (PDF) [file pone.0207640.s003.pdf]

S3. Differences in vertical distribution of three terrestrial hermit crab species.

Elevation(m)	*C. rugosus*(count)	*C. cavipes*	*C. brevimanus*
2	9		
3	20		
2	6		
3	2		
2	90		
2	2		
3	20	4	
2	63		
3	131		
2	71		1
2	30	8	2
3	8	1	1
2	84	2	
3	108		
2	93	1	
6		31	2
6	17		
13			
17			
19			
17			
18			3
6	21		
13			8
17			27
19			10
17			24
18			60
6	62		
13			
17			9
19		1	5
17			5
18			9
6			
5			

6	78		
7	72		
10	151		
6	5	1	1
5	27	2	
6	97		
7			
10	34		
6	3	4	
5			
6	125		
7			
10	53	1	1
