# Supplementary material for: What are the sympatric mechanisms for three species of terrestrial hermit crab (Coenobita rugosus, C. brevimanus, and C. cavipes) in coastal forests?
Source: PLoS One. 2018 Dec 12;13(12):e0207640. doi: 10.1371/journal.pone.0207640 (PMC6291072; doi:10.1371/journal.pone.0207640)
Supplement: S4 File — (PDF) [file pone.0207640.s004.pdf]

S4. Differences in shell preference of three species of terrestrial hermit crabs with overlapping body size (0.7–1.3 cm, shield length) and differences in shell size.

C. rugosus (97)	*C. cavipes* (21)	*C. brevimanus* (45)
1	1.65	1.6
1.2	1.1	1.5
1	2.2	1.05
1.2	1.85	1.49
1.3	1.4	1.15
1	1.3	1.2
1.21	2.2	1.05
1.3	2.3	1.1
1.35	2	1.45
1.4	2	1.3
1.3	2	1.2
1.4	1.95	1
1.2	1.7	1.2
1.2	1.55	1.1
1.1	1.85	1.69
1.2	2	1.53
1.3	1.85	1.6
1.35	1.3	1.3
1.4	2.3	1.05
1.4	2	1.45
1.5	1.95	1.2
1.48		1.25
1.5		1.08
1.5		1.26
1.85		1.29
1.35		1.4
1.6		1.5
1.5		1.35
1.65		1.5
1.7		1.2
1.5		1.3
1.7		2.2
0.6		1.38
1.5		1.5

1.5	2.3
1.5	2
1.6	1.5
1.3	1.92
1.45	1.55
1.5	1.55
1.6	1.8
1.6	2.3
1.75	2.1
1.75	2
1.85	2.6
1.2	
1.5	
1.71	
1.55	
1.7	
1.7	
1.9	
1.5	
1.7	
1.6	
1.8	
1.85	
2.1	
2.2	
1.69	
1.75	
2	
1.98	
1.73	
1.65	
1.8	
1.82	
1.92	
1.98	
2	
2	
2	

2
2
2
2
2.1
2.1
2.1
2
1.9
1.95
2
2
2.1
2
2
1.9
1.92
2
2
2.1
2.1
2.1
2.1
2.2
1.95
