# Supplementary material for: What are the sympatric mechanisms for three species of terrestrial hermit crab (Coenobita rugosus, C. brevimanus, and C. cavipes) in coastal forests?
Source: PLoS One. 2018 Dec 12;13(12):e0207640. doi: 10.1371/journal.pone.0207640 (PMC6291072; doi:10.1371/journal.pone.0207640)
Supplement: S5 File — (PDF) [file pone.0207640.s005.pdf]

S5. Differences in dietary preferences of three terrestrial hermit crab species

**Species /Food treatmeant**	**Banana**	**Saury**
C. rugosus	9	14
C. cavipes	8	2
C. brevimanus	2	9
